# Characterization of drilling waste from shale gas exploration in Central and Eastern Poland

**DOI:** 10.1007/s11356-018-2365-8

**Published:** 2018-05-28

**Authors:** Marzena Mikos-Szymańska, Piotr Rusek, Krzysztof Borowik, Maciej Rolewicz, Paulina Bogusz, Joanna Gluzińska

**Affiliations:** 1 Fertilizer Department, New Chemical Syntheses Institute, Al. Tysiąclecia Państwa Polskiego 13A, 24-110 Puławy, Poland; 2 New Chemical Syntheses Institute, Inorganic Chemistry Division “IChN” in Gliwice, Ul. Sowińskiego 11,, 44–101 Gliwice, Poland

**Keywords:** Drilling fluid, Cuttings, Microelements, Macroelements, Heavy metals, Recycling

## Abstract

The purpose of this research was to determine and evaluate the chemical properties of drilling waste from five well sites in Central and Eastern Poland. It was found that spent drilling fluids can contain high values of nickel and mercury (270 and 8.77 mg kg^−1^_,_ respectively) and can exceed the maximum permissible limits recommended by the EC regulations for safety of soils (75 mg kg^−1^ for nickel and 1.5 mg kg^−1^ for mercury). The heavy metal concentrations in the studied drill cuttings did not exceed the maximum permissible limits recommended by the EC regulation. Drilling wastes contain macroelements (e.g., calcium, magnesium, and potassium) as well as trace elements (e.g., copper, iron, zinc, and manganese) that are essential for the plant growth. It was stated that water extracts of drilling fluids and drill cuttings, according to anions presence, had not any specific constituents of concern based on FAO irrigation guidelines, the USEPA WQC, and toxicity values. X-ray diffraction analysis was used to understand the structure and texture of waste drilling fluid solids and drill cuttings. Analysis of the mineralogical character of drilling fluid solids revealed that they contained calcite, quartz, muscovite, sylvite, barite, dolomite, and orthoclase. Drill cuttings contained calcite quartz, muscovite, barite, dolomite, and barium chloride.

## Introduction

Drilling fluids (drilling muds) are one of the primary wastes generated from drilling operations. They are used to lubricate and the cool drilling apparatus, transport drill cuttings to the surface, and seal porous geologic formation (Yao and Naeth 2014; Fink 2011). Drilling fluids are made up of a base fluid (water, diesel or mineral oil, or a synthetic compound), weighting agents (e.g., barium sulphate), bentonite clay, lignosulphonates and lignites, and various additives that serve specific functions. Bentonite clay is used in drilling fluids to remove cuttings from the well and to form a filter cake on the walls of the hole, while lignosulphonates and lignites are used to keep the mud in a fluid state. Drilling fluid can contain toxic substances and are therefore considered environmentally damaging (Fink 2011; Drilling Waste Management Information System 2017). Drill cuttings are produced as the rock is broken by the drill bit advancing through rock or soil. They are made up of ground rock coated with a layer of drilling fluid.

Few studies have addressed the impact of disposal of spent drilling fluids on soil-plant-water systems. Some researchers found that high soluble salts, heavy metals, and petroleum residue contents in drilling fluids were detrimental to soil quality and plant growth (McFarland et al. 1994; Wojtanowicz 2008; Zvomuya et al. 2011). Others found positive or no impact from drilling fluid applied at low rates in coarse-textured soils in arid regions due to pH value increases, potential micronutrient addition, and improved soil properties (Lesky et al. 1989; Bauder et al. 2005; Yao and Naeth 2014, 2015). Few studies have focused on the release of toxic elements from oil well drill cuttings and their effect on soil and aquatic ecosystems (Magalhães et al. 2014; Purser and Thomsen 2012).

The management technologies and practices for drilling waste can be grouped into three major categories: waste minimization, recycle/reuse, and disposal. The volume of drilling waste released into the environment should be reduced for example by directional drilling that generates smaller volume of cuttings compared to the conventional one or by the use of techniques that need less drilling fluids and use alternative clean energy (solar, hydro, wind) for running drilling activities (Sharif et al. 2017). Recycling involves the conversion of wastes into usable materials that can be used to make new products. The waste can be used as substitutes for commercial products or as a feedstock in industrial processes (Zhang et al. 2016; Sharif et al. 2017). Disposal is the least preferred waste management option from the environmental point of view. Cuttings’ reinjection (Shadizadeh et al. 2011), onsite burial (Onwukwe and Nwakaudu 2012), waste pits, landfills, land-farming/land-spreading (Saint-Fort and Ashtani 2013), bioremediation, composting (Paladino et al. 2016), and vermi-culture (Adekomaya 2014; Sharif et al. 2017) are the examples of disposal methods for onshore operations.

In Poland, the first borehole, aimed at the exploration of natural gas from shales, was drilled in the year 2010. Natural gas from shale accumulations is released through drilling holes reaching depths of several thousand meters. Hydraulic fracturing operations generate a considerable amount of waste (Pyssa 2016).

The purpose of this research was to determine and evaluate the chemical properties of drilling waste from shale gas drilling activities in Central and Eastern and South-Eastern Poland. The objectives of this research were (i) contrast chemical characteristics of drilling waste samples; (ii) identify specific constituents of concern (COCs) and differences in anion concentrations in water extracts of drilling waste by comparing them with FAO guidelines for agriculture uses, USEPA water quality criteria for surface discharge, and toxicity values for *D. magna* and *P. promelas*; and (iii) to determine the mineralogical compositions of drilling fluid solids and drill cuttings.

## Material and methods

### Samples

The object of analysis covers Silurian and Ordovician shale formations in Poland (Porębski et al. 2013; Jarzyna et al. 2017). Samples of the spent bentonite potassium drilling fluid and drill cuttings were collected from well sites located in Central and Eastern Poland in 2015–2016. Samples came from five different locations of drilling sites. Drilling fluids and drill cuttings were collected as two separate samples. Samples of drilling fluid and cuttings (DF1 and C1, respectively) were from Dobryniów in Lublin Vivodeship, Kościaszyn in Lublin Vivodeship (DF2 and C2, respectively), Przemyśl in Subcarpathian Voivodeship (DF3 and C3, respectively), Lubliniec in Subcarpathian Voivodeship (DF4 and C4, respectively), and Łochów in Masovian Vivodeship (DF5 and C5, respectively) shale gas drilling concessions.

### Sample preparation

A collected sample of drilling fluid was dried at 50 °C to obtain a solid; afterwards, it was grounded and homogenized. A drill cuttings’ sample was dried in a laboratory oven at 105 °C, in the amount of 1.2 kg. The dried sample was preliminary crushed and then grounded using a laboratory ham mill.

For XRD analysis, a collected drilling fluid, in suspension, was dried at 50 °C in order to obtain solids. Cuttings were dried in a laboratory oven at 105 °C in order to obtain a solid. Dried samples were crushed in a porcelain mortar and sieved to obtain a homogenous powder with grains under 50 μm.

### Analytical methods

Analytical methods, used for determination of metal contents in drilling fluid and cuttings samples by ICP-OES and mercury content by CV-AAS, are described in previous article (Gluzińska et al. 2017).

### Ion chromatography

#### Equipment

Chloride and sulphate analyses in drilling wastewater extracts were conducted using an ion chromatograph ICS-3000 (Dionex Company) working in an external water mode. Chromeleon 6.7 Chromatography Management Software (Dionex) was used for the system control and data processing.

#### Reagents and solutions

Multi-Component Anion Mix 4, (F^−^, Br^−^, Cl^−^, PO_4_^3−^, NO_3_^−^, SO_4_^2−^, c = 100 μg/ml) (Acculon) as a reference standard for quantitative determination of studied anions was used. Water, 18.2 MΩ WaterPro PS Labconco, free of particles of diameter > 0.2 μm was used. As an eluent, 30 Mm NaOH (Fluka; sodium hydroxide; puriss. P.a. ACS; ≥ 98.0%; pellets) was used. Calibration standard solutions for those ions determinations were prepared from the standard solution by dissolution with deionized water. A calibration concentration range for determined ions was 0.1; 0.5; 2; 5 mg/dm^3^.

#### Sample preparation

Samples of water extracts of drilling waste, weighing about 1–2 g, were dissolved in water in a 250-ml volumetric flask and was made up to the mark. An analytical sample was prepared from the solution by dilution with water in a ratio 1:100. Analysis was carried out in three parallel repetitions. The diluted sample was passed through a 0.45-μm membrane filter just before injection to the chromatographic column.

#### Chromatograph operating conditions

Conditions of carried out chromatographic analysis are presented in Table 1.

**Table 1:** Chromatographic analysis conditions

Analytical column + guard column	AS11-HC (4 × 250 mm) + AG11-HC (4 × 50 mm)
Eluent	30 Mm NaOH
Eluent flow rate	1.5 ml/min
Pressure in the column	~ 1940 psi
Injection volume	25 μl
Column operating temperature	30 °C
Conductometer cell temperature	35 °C
Suppression type	ASRS 300–4 mm
Suppressor current intensity	112 Ma
Detection	Conductometric
Analysis time	10 min

### Comparison of anions in water extracts of drilling waste with water use criteria

A risk-based approach was used to identify specific constituents of concern (COCs) in the water extracts of drilling fluid solids and water extracts of cuttings. COCs were identified as anions in water extracts of drilling solid waste at sufficient concentrations to pose potential risks to receiving system biota and crops. Comparison of anion concentrations to the Food and Agriculture Organization of the United Nations (FAO) numeric standards for irrigation, United States Environment Protection Agency (USEPA) water quality criteria (WQC), and toxicity values for *D. magna* and *P. promelas* was used to discern COCs in the water extracts of drilling waste solids (Ayers and Westcot 1994; USEPA 2007).

### Powder X-ray diffraction

The XRD measurements of samples were performed on a PANanalytical Empyrean system (Bragg-Brentano geometry) equipped with a PIXcel^3D^ detector using Cu Kα radiation (*λ* = 1.542 Å) and operating at 40 Kv and 40 Ma. The samples were scanned between 10 < 2θ < 70°, with the step size 0.01° 2θ and time/step 30 s. The quantity analysis of crystallographic phases was automatic using Rietveld’s method with Brindley corrections for micro-absorption and manual corrections of results for better fitting parameters. The line broadening was determined in the High-Score Plus software. The pseudo-Voigt function for peak size approximations was used.

## Results and discussion

### Metal contents by ICP-OES and mercury content by CV-AAS

**Fig. 1:**
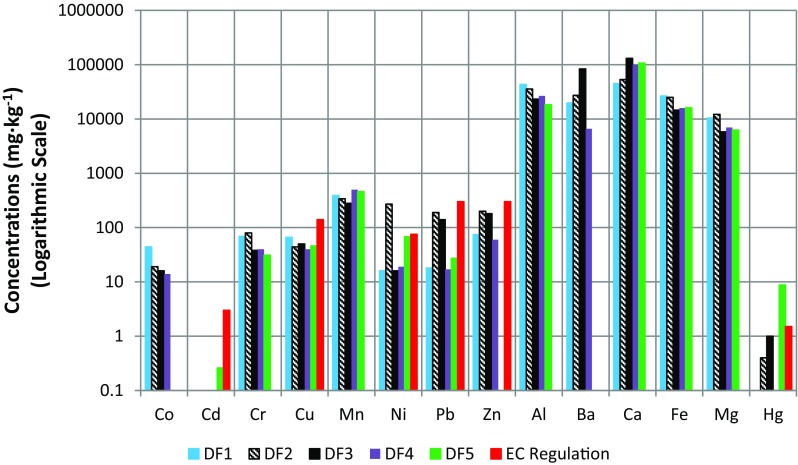
Concentrations of metals in drilling fluids from five different drilling locations in Poland

The chemical characteristics of drilling waste depend largely on geological factors related to the shales deposits and on different drilling techniques used at the well sites, namely the type of muds (e.g., water-, oil-, or synthetic-based muds), and the method of drilling (e.g., traditional or pneumatic). Thus, drilling waste from every drilling activities has its own chemical characteristics. The elemental composition of drilling waste was determined by ICP-OES and mercury (Hg) by CV-AAS. Elements, such as cobalt (Co), cadmium (Cd), chromium (Cr), copper (Cu), manganese (Mn), nickel (Ni), lead (Pb), zinc (Zn), aluminum (Al), barium (Ba), calcium (Ca), iron (Fe), magnesium (Mg), and mercury (Hg) in drilling fluid and drill cuttings samples were detected. Ca was the most predominant element in drilling waste with the concentration exceeding 50 g kg^−1^. Ca content in drilling fluid ranged from 53.4 to 131 g kg^−1^ (Table 1). In other study, Ca level in drilling wastes (oil-based fluids and cuttings) was on average 87.3 g kg^−1^ (Adekunle et al. 2013). Co content in drilling fluids ranged from 14 to 44 mg kg^−1^. Kisic et al. (2009) conducted survey of 20 drilling fluid samples taken from the central waste pit of oil/gas fields. In case of heavy metals, the samples contained elements such as Cd, Hg, Pb, As, Ni, Cu, Cr, Zn, Ba, and Ca that contained on average 9.6, 3.8, 219, 41.2, 34.3, 31.2, 57.8, 206, and 2373 mg kg^−1^, and 9.03 g kg^−1^, respectively. The level of Cd in drilling fluids was significantly lower (0–0.26 mg kg^−1^) compared to that determined in the study (8.8–11.0 mg kg^−1^ of Cd). A maximum level of Pb in the drilling fluid (190 mg kg^−1^) was similar to the average content of Pb in the study (Kisic et al. 2009). In our study, Ni content in drilling fluids ranged from 16 to 270 mg kg^−1^ whereas in the samples evaluated by Kisic et al. (2009) ranged from 27.5 to 39.5 mg kg^−1^. Cu content in studied drilling fluids ranged from 40 to 66 mg kg^−1^ whereas Cu content in drilling fluid samples ranged from 26.8 to 41.6 mg kg^−1^ in the mentioned study. Cr and Zn contents in drilling fluids were similar (31–80 and 60–200 mg kg^−1^, respectively) to those contents in drilling fluids from waste pit in Croatia (47.2–68.2 mg kg^−1^ of Cr and 139–295 mg kg^−1^ of Zn) (Kisic et al. 2009). Drilling waste from other well sites in Poland contained toxic heavy metals such as Cr (17.2–35.6 mg kg^−1^), Ni (22.9–46.8 mg kg^−1^), Zn (31.6–276.0 mg kg^−1^), Pb (11.5–211.5 mg kg^−1^), and Cu (28.3–160.1 mg kg^−1^) (Śliwka et al. 2012). Moreover, Śliwka et al. (2012) stated that the majority of samples (five from eight drilling waste samples) did not exceed dangerous level of total content of toxic heavy metals for soil environment (150 mg kg^−1^ d m). Research conducted by Steliga and Uliasz (2014) showed that bentonite drilling fluids after a coagulation contained 985 mg kg^−1^ of Ba, 201.9 mg kg^−1^ of Pb, 86.6 mg kg^−1^ of Cu, 25.3 mg kg^−1^ of Cr, 20.1 mg kg^−1^ of Ni, and 1.8 mg kg^−1^ of Hg. Figure 1 shows metal contents in drilling fluids from five different drilling locations. Taking into account, the EC Regulation No. 86/278/EEC, only one of the five samples (DF2) exceeded the maximum permissible limit values (30–75 mg kg^−1^) for Ni content (270 mg kg^−1^) and one of the five samples (DF5) contained a high amount of Hg (8.77 mg kg^−1^) comparing to limit values for soils (1–1.5 mg kg^−1^) (the PL Regulation of the Minister of Environment 2016).

The characteristics of drill cuttings, including metal concentrations, are showed in Table 3. They contain macroelements (Ca, Mg, K, Na), microelements (Cu, Co, Fe, Mn, Zn, As, Al, Ba), and heavy metals (Cr, Cd, Pb, Ni, Hg). Ca content in cuttings ranged from 51.1 to 116 g kg^−1^, Mg content ranged from 8.19 to 20.2 g kg^−1^, and K content ranged from 16.0 to 34.0 g kg^−1^ (Table 3). Na content was determined only in the one drill cuttings sample and it was 3.68 g kg^−1^. Comparing the concentrations of contaminants in drill cuttings with research results obtained by other researchers in the world (Table 4), it was stated that Al contents in drill cuttings (30,600 to 62,500 mg kg^−1^) were higher than in cuttings from Brazil (23,000 mg kg^−1^) (Junior et al. 2017). As content in drill cuttings (C5) was 8.1 mg kg^−1^ and in cuttings from the USA (Leonard and Stegemann 2010) and Nigeria (Kogbara et al. 2016) was 5 and 10.8 mg kg^−1^, respectively. Ba contents in drill cuttings ranged from 8600 to 81,400 and the drill cuttings from Brazil (Junior et al. 2017) and from the USA (Leonard and Stegemann 2010) contained 18,000 and 51,500 mg kg^−1^ of Ba, respectively. Cd contents (0–0.4 mg kg^−1^) were smaller than those in cuttings from the USA (21 mg kg^−1^) (Leonard and Stegemann 2010). Co content in cuttings ranged from 12 to 27 mg kg^−1^ and in the cuttings from the USA, it was 14 mg kg^−1^. In our study, Cr contents in drill cuttings ranged from 72 to 140 mg kg^−1^, and in the study conducted by Kujawska and Cel (2017), they contained 65.76 mg kg^−1^ of Cr. The ranges of Cr contents were similar to the Cr content in drill cuttings from the USA (106 mg kg^−1^) and higher than Cr contents in cuttings from Nigeria (0.01 to 0.65 mg kg^−1^) (Gbadebo et al. 2010; Kogbara et al. 2016). Cu contents in cuttings ranged from 41 to 85 mg kg^−1^ and were similar to those in cuttings from the USA (44 mg kg^−1^). They were higher than Cu contents in cuttings from Nigeria (0–0.16 mg kg^−1^) (Gbadebo et al. 2010). Kogbara et al. (2016) determined the Cu content in drill cuttings from Nigeria and they contained 114 mg kg^−1^ of Cu. In our study, Mn contents in cuttings ranged from 410 to 730 mg kg^−1^, drill cuttings from the USA contained 345 mg kg^−1^ of Mn, whereas drill cuttings from Nigeria contained very small amounts of Mn (0.26–3.45 mg kg^−1^). It was stated that drill cuttings from Polish well sites were characterized by the higher ranges of Ni, Pb, and Zn content (24–70.1, 28.1–250, and 66–160 mg kg^−1^, respectively) compared to drill cuttings from Nigeria (0–2.12, 0–2.19, and 0.02–0.55, respectively) (Gbadebo et al. 2010). The study of contaminant contents in drill cuttings from Nigeria conducted by Kogbara et al. (2016) revealed that they contained 10.5 mg kg^−1^ of Ni, 178 mg kg^−1^ of Pb, and 196 mg kg^−1^ of Zn. Studies of metal contents in drill cuttings from the USA (Leonard and Stegemann 2010) showed that they contained 38 mg kg^−1^ of Ni, 150 mg kg^−1^ of Pb, and 82 mg kg^−1^ of Zn. The mentioned researchers did not determine the Hg contents in drill cuttings.

**Fig. 2:**
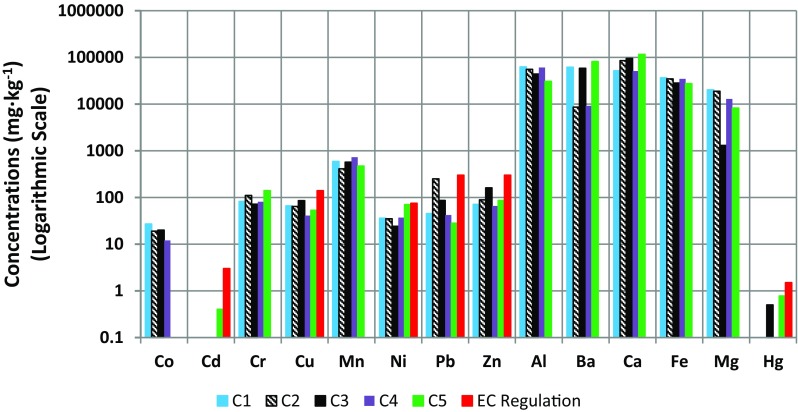
Concentrations of metals in drill cuttings from five different drilling locations in Poland

The results showed the trend of higher metal contents such as Mg, Cu, Fe, Mn, Zn, Al, Cr, Pb, and Ni in the drill cuttings than in drilling fluids and it was confirmed by other researchers (Gbadebo et al. 2010; Veritas 2000) (Figs. 1 and 2; Tables 2 and 3). To determine and identify specific metal species and their binding forms in drill cuttings, the chemical fractionation should be used. The heavy metals and nutrients mobility are connected with the solubility of their forms. The sequential extraction analysis can be used in order to determine the mobility of metals from drill cuttings. In the Community Bureau of Reference (BCR) method procedure, the following fractions can be distinguished: exchangeable (the most mobile metals), reducible (elements absorbed or co-precipitated with Fe and Mn oxides, medium mobility), oxidizable (metals bound to organic matter, medium mobility), and residual (metals strongly bound to the solid phase). According to research conducted by Kujawska and Cel (2017), heavy metals in drill cuttings were mainly bound to the organic fraction. Stuckman et al. (2016) also stated that metals present in drilling cuttings such as Cu, Ni, Zn, Cd, and Co were mainly associated with oxidizable phases. It can be stated that these metals present in drill cuttings are characterized by medium mobility. Moreover, drill cuttings are made of ground rock while drilling fluids contain mainly water elements that could be extracted from rock or soil during drilling operations and substances that were added to compose their formulations. Figure 2 shows metal contents in drill cuttings from five different drilling locations. Taking into account, the Council Directive 86/278/EEC for heavy metal maximum permissible limits for soils, the studied drill cuttings samples did not exceed these limits. One of the drill cuttings sample (C2) exceeded the maximum permissible limit for Pb content recommended by the Regulation of the Minister of Environment (Poland) (2016), for safety of the I class of soil. Mostavi et al. (2015) compared the results of drill cuttings chemical analysis with the Toxicity Characteristic Leaching Procedure regulatory levels and stated that examined drill cuttings can be classified as non-hazardous waste.

**Table 2:** Chemical analysis of drilling fluids (Central and Eastern Poland, 2015–2016)

Metal contents	Method	DF1	DF2	DF3	DF4	DF5	PL Regulation*)				EC Regulation**)
							I	II	III	IV	Soils; pH = 6–7
Macroelements											
Ca (g kg^−1^)	ICP-OES	44.7 ± 6.7	53.4 ± 8.0	131.0 ± 19.6	99.6 ± 14.9	100.7 ± 15.2					
Mg (g kg^−1^)	ICP-OES	10.7 ± 1.6	12.2 ± 1.8	5.8 ± 0.9	7.0 ± 1.1	6.25 ± 0.9					
K (g kg^−1^)	ICP-OES	14.7 ± 2.2	53.5 ± 8.0	53.2 ± 8.0	59.7 ± 9.0	60.4 ± 9.1					
Na (g kg^−1^)	ICP-OES	–	–	–	–	11.4 ± 1.7					
Microelements											
Cu (mg kg^−1^)	ICP-OES	66.0 ± 9.9	44.0 ± 6.6	50.0 ± 7.5	40.0 ± 6.0	45.8 ± 6.9	200	100–300	300	600	50–140
Co (mg kg^−1^)	ICP-OES	44.0 ± 6.6	19.0 ± 2.9	16.0 ± 2.4	14.0 ± 2.1	–					
Fe (g kg^−1^)	ICP-OES	26.5 ± 4.0	25.0 ± 3.8	14.6 ± 2.2	15.9 ± 2.4	16.1 ± 2.4					
Mn (mg kg^−1^)	ICP-OES	390.0 ± 58.5	340.0 ± 51.0	280.0 ± 42.0	500.0 ± 75.0	460.0 ± 69.0					
Zn (mg kg^−1^)	ICP-OES	74.0 ± 11.1	200.0 ± 30.0	180.0 ± 27.0	60.0 ± 9.0	78.9 ± 11.8					
As (mg kg^−1^)	ICP-OES	–	–	–	–	8.05 ± 1.2	25	10–50	50	100	
Al (g kg^−1^)	ICP-OES	43.3 ± 6.5	35.6 ± 5.3	23.3 ± 3.5	26.7 ± 4.0	18.3 ± 2.7					
Ba (g kg^−1^)	ICP-OES	19.6 ± 2.9	27.2 ± 4.0	83.6 ± 12.5	66.0 ± 9.9	61.1 ± 12.2***)	0.4	0.2–0.6	1.0	1.5	
Heavy metals											
Cr (mg kg^−1^)	ICP-OES	69.0 ± 10.4	80.0 ± 12.0	38.0 ± 5.7	40.0 ± 6.0	31.0 ± 4.6	200	150–500	500	1000	
Cd (mg kg^−1^)	ICP-OES	< 0.005	< 0.005	< 0.005	< 0.005	0.26 ± 0.04	2	2–5	10	15	1–3
Pb (mg kg^−1^)	ICP-OES	18.0 ± 2.7	190.0 ± 28.5	140.0 ± 21.0	17.0 ± 2.6	27.1 ± 4.1	200	100–500	500	600	50–300
Ni (mg kg^−1^)	ICP-OES	16.0 ± 2.4	270.0 ± 40.5	16.0 ± 2.4	19.0 ± 2.9	67.8 ± 10.2	150	100–300	300	500	30–75
Hg (mg kg^−1^)	CV-AAS	0.10 ± 0.02	0.40 ± 0.09	1.00 ± 0.22	–	8.77 ± 1.93	5	2–5	10	30	1–1.5

*)Maximum permissible limits of heavy metal contents in soils (depth 0–0.25 m below the ground level) for I–IV class of soil according to the Regulation of the Minister of Environment (Poland), 2016

**)Maximum permissible limits for heavy metal contents in soils, pH = 6–7 according to Directive 86/278/EEC

***)A total Ba content determined by XRD

–Not determined

**Table 3:** Chemical analysis of drill cuttings (Central and Eastern Poland, 2015–2016)

Metal contents	Method	C1	C2	C3	C4	C5	PL Regulation*)				EC Regulation**)
							I	II	III	IV	Soils; pH = 6–7
Macroelements											
Ca (g kg^−1^)	ICP-OES	51.7 ± 7.8	85.3 ± 12.8	95.2 ± 14.3	51.1 ± 7.7	116.0 ± 17.4					
Mg (g kg^−1^)	ICP-OES	20.2 ± 3.0	18.8 ± 2.8	13.0 ± 0.2	12.9 ± 1.9	8.19 ± 1.2					
K (g kg^−1^)	ICP-OES	24.0 ± 3.6	34.0 ± 5.1	27.2 ± 4.1	29.1 ± 4.4	16.0 ± 2.4					
Na (g kg^−1^)	ICP-OES	–	–	–	–	3.68 ± 0.6					
Microelements											
Cu (mg kg^−1^)	ICP-OES	66.0 ± 9.9	64.0 ± 9.6	85.0 ± 12.8	41.0 ± 6.2	53.0 ± 8.0	200	100–300	300	600	50–140
Co (mg kg^−1^)	ICP-OES	27.0 ± 4.1	19.0 ± 2.9	20.0 ± 3	12.0 ± 1.8	–					
Fe (g kg^−1^)	ICP-OES	36.5 ± 5.5	34.7 ± 5.2	28.2 ± 4.2	34.7 ± 5.2	27.3 ± 4.1					
Mn (mg kg^−1^)	ICP-OES	590.0 ± 88.5	410.0 ± 61.5	570.0 ± 85.5	730.0 ± 109.5	470.0 ± 70.5					
Zn (mg kg^−1^)	ICP-OES	71.0 ± 10.7	89.0 ± 13.4	160.0 ± 24.0	66.0 ± 9.9	86.1 ± 12.9					
As (mg kg^−1^)	ICP-OES	–	–	–	–	8.1 ± 1.2	25	10–50	50	100	
Al (g kg^−1^)	ICP-OES	62.5 ± 9.4	55.6 ± 8.3	52.5 ± 7.9	60.7 ± 9.1	30.6 ± 4.6					
Ba (g kg^−1^)	ICP-OES	61.6 ± 9.2	8.6 ± 1.3	58.4 ± 8.8	9.2 ± 1.4	81.4 ± 16.3***)	0.4	0.2–0.6	1.0	1.5	
Heavy metals											
Cr (mg kg^−1^)	ICP-OES	82.0 ± 12.3	110.0 ± 16.5	72.0 ± 10.8	81.0 ± 12.2	140.0 ± 21.0	200	150–500	500	1000	
Cd (mg kg^−1^)	ICP-OES	< 0.005	< 0.005	< 0.005	< 0.005	0.4 ± 0.1	2	2–5	10	15	1–3
Pb (mg kg^−1^)	ICP-OES	45.0 ± 6.75	250.0 ± 37.5	86.0 ± 12.9	42.0 ± 6.3	28.1 ± 4.2	200	100–500	500	600	50–300
Ni (mg kg^−1^)	ICP-OES	36.0 ± 5.4	35.0 ± 5.3	24.0 ± 3.6	37.0 ± 5.6	70.1 ± 10.5	150	100–300	300	500	30–75
Hg (mg kg^−1^)	CV-AAS	–	0.10 ± 0.02	0.50 ± 0.11	–	0.77 ± 0.17	5	2–5	10	30	1–1.5

*)Maximum permissible limits of heavy metal contents in soils (depth 0–0.25 m below the ground level) for I–IV class of soil according to the Regulation of the Minister of Environment (Poland), 2016

**)Maximum permissible limits for heavy metal contents in soils, pH = 6–7 according to Directive 86/278/EEC

***)A total Ba content determined by XRD

–Not determined

**Table 4:** Comparison of the range of contaminant concentrations in the drill cuttings with research data conducted by other researchers

Contaminants	Metal contents in drill cuttings	Kujawska and Cel (2017)	Junior et al. (2017)	Leonard and Stegemann (2010)	Gbadebo et al. (2010)	Kogbara et al. (2016)
	Poland	Poland	Brazil	USA	Nigeria	Nigeria
Al (mg kg^−1^)	30,600–62,500	–	23,000	–	–	–
As (mg kg^−1^)	8.1	–	–	5	–	10.8
Ba (mg kg^−1^)	8600–81,400	1911.33	18,000	51,500	–	–
Cd (mg kg^−1^)	0–0.4	–	–	21	–	–
Co (mg kg^−1^)	12–27	0.2	–	14	–	–
Cr (mg kg^−1^)	72–140	65.76	–	106	0.01–0.65	0.22
Cu (mg kg^−1^)	41–85	104.29	–	44	0–0.16	114
Mn (mg kg^−1^)	410–730	469	–	345	0.26–3.45	–
Ni (mg kg^−1^)	24–70.1	21.75	–	38	0–2.12	10.5
Pb (mg kg^−1^)	28.1–250	41.92	–	150	0–2.19	178
Hg (mg kg^−1^)	0.1–0.774	–	–	–	–	–
Zn (mg kg^−1^)	66–160	62.1	–	82	0.02–0.55	196
Fe (mg kg^−1^)	27,300–36,500	14,370	27,000	26,400	1.95–714	–

–Not determined

**Table 5:** Comparison of the range of anions in water extracts of drilling waste with research data conducted by other researchers and with irrigation guidelines, surface water discharge criteria (SDW), and toxicity values for *D. magna* and *P. promelas*

Samples	Anions (mg kg^−1^)					
	Br^−^	Cl^−^	F^−^	NO_3_^−^	PO_4_^3−^	SO_3_^2−^
DF5	ND	24.40	ND	ND	ND	0.87
C5	ND	5.70	ND	ND	ND	6.37
HFWE^a^	851	75,100	ND	ND	–	199
WP^a^	15.9	9000	ND	ND	–	2600
SGPW^b^	ND-10600	48.9–212,700	ND-33	ND-2670	ND-5.3	ND-3663
TGSPW^b^	–	52–216,000	–	–	–	12–48
CBMPW^b^	0.002–300	0.7–70,100	0.05–15.22	0.002–18.7	0.05–1.5	0.01–5590
NGPW^b^	0.038–349	1400–190,000	–	–	–	1.0–47
Water use criteria						
Irrigation		1050	1	10	2	960
SDW		230		10	0.025	
Toxicity values LC50	2.7 (15 d Dm^c^)	7341 (96 h Pp^d^)	315 (96 h Pp^e^)	1341 (96 h Pp^f^)	100 (96 h Pp^g^)	
COCs*	No	No	No	No	No	No

*HFWE* hydraulic fracturing well effluent, *PW* wastewater from pit, *SGPW* shale gas produced water, *TGSPW* tight gas sand produced water, *CBMPW* coalbed methane produced water, *NGPW* conventional natural gas produced water, *ND* not detected, – not determined, *COCs are defined as constituents in water extracts of drilling waste that have concentrations in excess of the use guidelines, *Dm Daphnia magna*, *Pp Pimephales promelas*

^a^Thacker et al. (2015). ^b^Alley et al. (2011). ^c^Canton and Wegman (1983). ^d^Mount et al. (1997). ^e^Smith et al. (1985). ^f^Scott and Crunkilton (2000). ^g^Ewell et al. (1986)

**Table 6:** Minerals in drilling waste samples

Minerals	Drilling fluid solids (%)	Drill cuttings (%)	Drill cuttings (%)		Drilling fluid dried powder solids (%)
			Wilke et al. (2015)		Sawaengpol and Wannakomol (2017)
	Silurian and Ordovician shale, Poland		Upper Cambrian Alum shale, Denmark	Lower Jurassic Posidonia shale, Germany	Thailand
Quartz	24.6	29.2	–	8.9–25.6	43.83
Barite	10.4	13.3	–	5.1	1.39
Calcite	35.8	43.6	–	–	14.21
Dolomite	2.8	3.4	–	–	–
Sylvite	11.0	–	–	–	–
Muscovite-2M1	12.7	10.1	41.1–43.7*^)^	7.0–25.5*^)^	–
Orthoclase	2.7	–	6.3	–	–
Barium chloride	–	0.5	–	–	–
Carbonate	38.6**^)^	47.0**^)^	–	30.3–73.4	–
Kaolinite	–	–	–	2.8–30.3	32.82
Pyrite	–	–	10.7–11.4	2.5–8.7	–
Jarosite	–	–	4.2	–	–
Sanidine	–	–	8.9	–	–
Albite	–	–	–	4–5.4	7.74

*Muscovite/illite, **Carbonate (calcite+dolomite)

Despite the fact that the total Ba levels in studied samples were high (6.6–83.6 and 8.6–81.4 g kg^−1^ for drilling fluids and drill cuttings, respectively), this element existed mainly in the BaSO_4_ form which is a water and acid insoluble, and in this form, the barium compound does not pose a threat to the environment. For comparison, the maximum permissible limits for heavy metal levels in soils according to Polish law are presented (Table 2). It has been observed that drilling wastes are relatively rich in calcium, magnesium, and potassium which are differently required by different species of plants and animals in the soil and water environment. Similar results were obtained in the research conducted on both the oil-based and water-based drilling wastes from Nigerian wells (Gbadebo et al. 2010).

Following the chemical characterization of the drilling fluids and drill cuttings, the main contaminants were found to be Ba, Ni, Mn, Cu, Cr, Pb, arsenic (As), and Hg.

### Ion chromatography (IC)

**Fig. 3:**
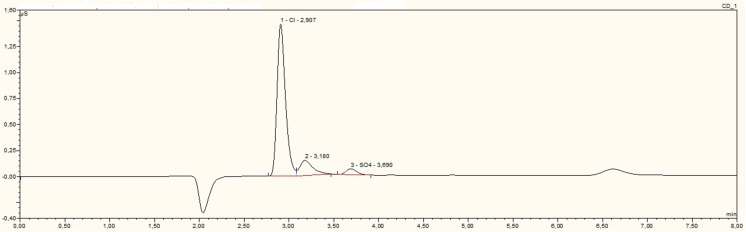
An anionic chromatogram of the water extract of drilling fluid (DF5)

**Fig. 4:**
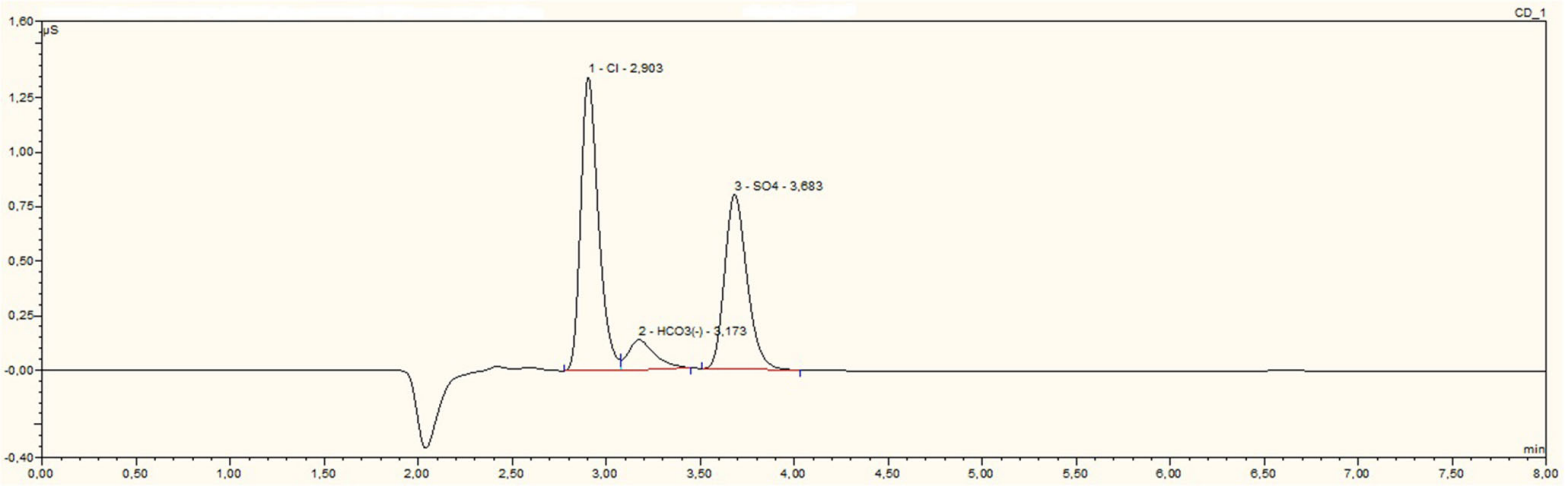
An anionic chromatogram of the water extracts of drill cuttings (C5)

Figures 3 and 4 show chromatograms of water extracts of drilling fluid and drill cuttings. Among determined ions, only chloride (R_t_ = 2.9 ± 0.1 min) and sulphate (Rt = 3.69 ± 0.1 min) were identified. In an anionic chromatogram of water extracts of drilling waste, carbonate peaks were identified (R_t_ = 3.18 ± 0.1 min). In the chromatogram of water extracts of drilling fluid is an unidentified peak with a retention time of 6.6 min. Quantity analysis showed that water extracts of drilling fluid contained 24.4 mg kg^−1^ of chloride and 0.87 mg kg^−1^ of sulphate. Water extracts of drill cuttings contained 5.7 mg kg^−1^ of chloride and 6.37 mg kg^−1^ of sulphate. F^−^, Br^−^, PO_4_^3−^, and NO_3_^−^ ions were not identified in studied water extract samples of drilling waste.

The values of anion concentrations both in the water extracts of drilling fluid solids and drill cuttings are lower than those reported by other researchers (Alley et al. 2011; Thacker et al. 2015; Canton and Wegman 1983; Ewell et al. 1986; Mount et al. 1997; Scott and Crunkilton 2000; Smith et al. 1985) for other types of produced water. Beneficial use criteria were compared to anion concentrations (Br^−^, Cl^−^, F^−^, NO_3_^−^, PO_4_^3−^, SO_3_^2−^) to discern COCs present in water extracts of drilling fluid solids and water extracts of drill cuttings. It was stated that these water extracts, according to anions presence, had no COCs based on FAO irrigation guidelines, the USEPA WQC and toxicity values (Table 5).

### XRD analysis

**Fig. 5:**
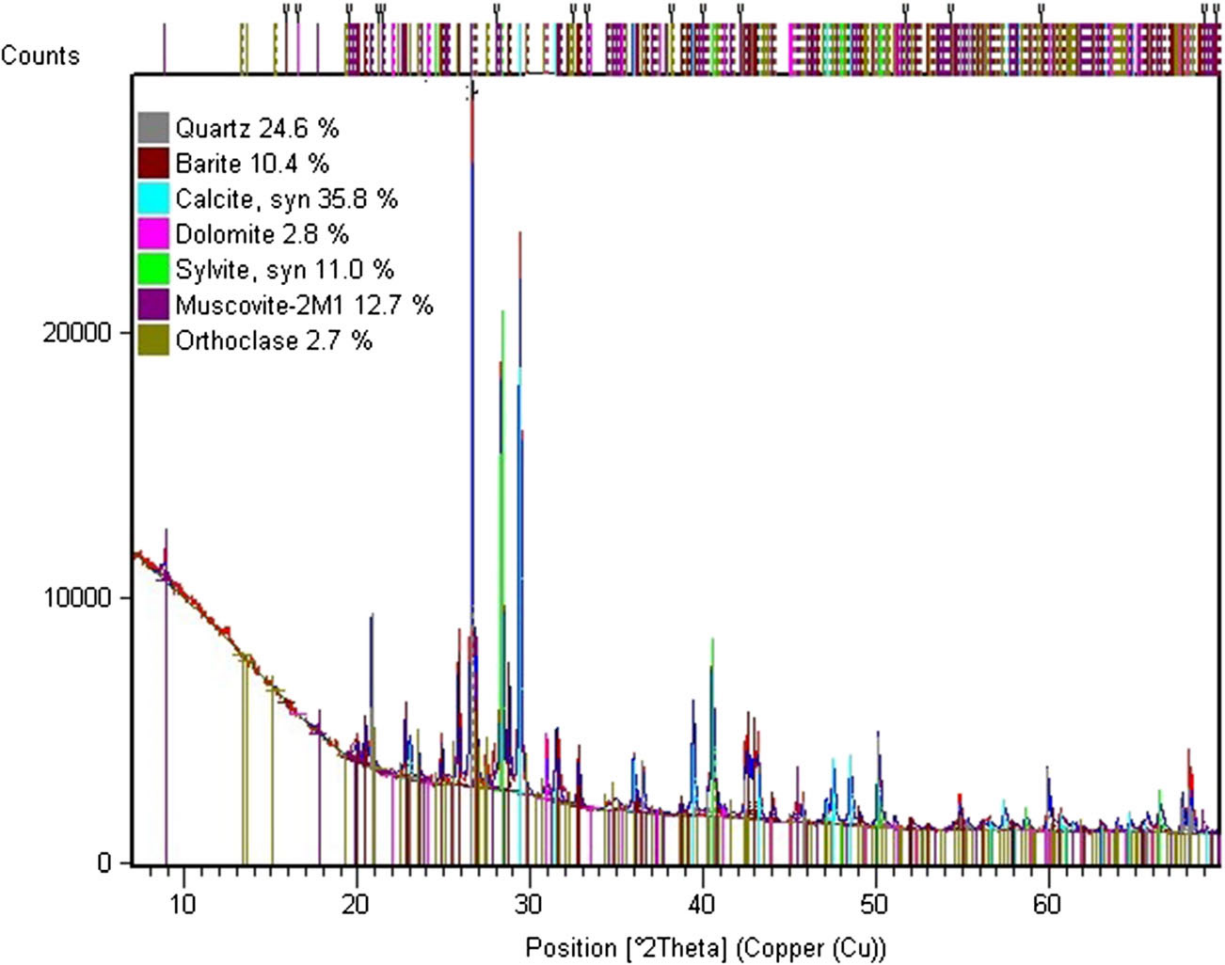
XRD pattern of drilling fluid solids (DF5) dried at 50 °C

**Fig. 6:**
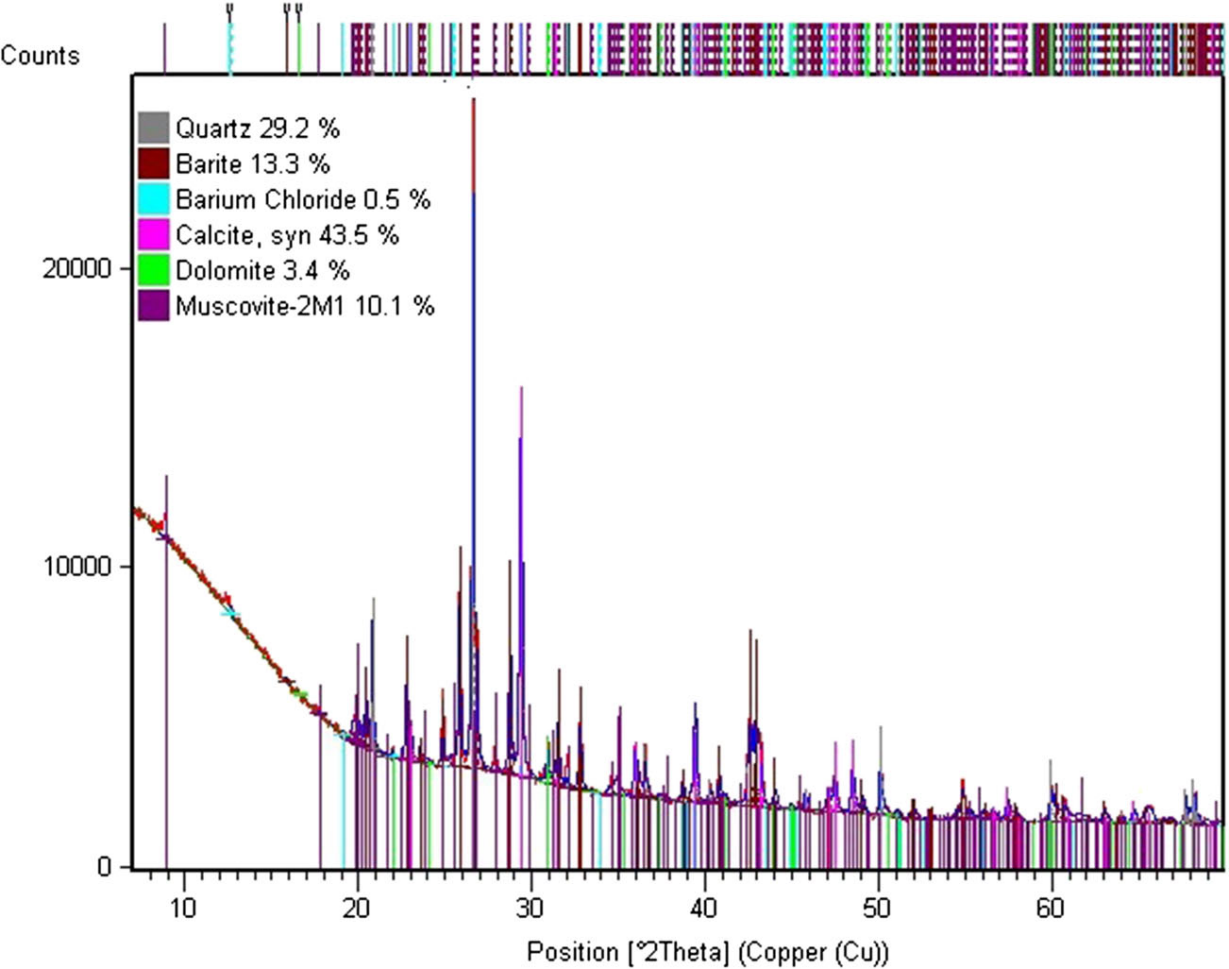
XRD pattern of cuttings sample (C5) dried at 105 °C

XRD analysis was used to identify crystalline compounds (mineral) based on their crystal structure. Each compound gives a unique pattern of diffraction peaks. Both drilling fluid solids and drill cuttings are characterized by very complex phase compositions. Rietveld’s analysis showed a very good fitting of a model and an experimental diffraction pattern. A result of the study is quality and semi-quantity analysis of compounds occurring in a crystalline form which are summed up to 100%. Phases of amorphous, organic, and other compounds that are present in trace amounts in the samples are not taken into consideration in the balance, which means that the real element contents in studied samples are slightly lower. X-ray powder diffraction patterns of drilling waste are shown in Figs. 5 and 6.

Analysis of the mineralogical character of drilling fluid solids (DF5) revealed that they contained 35.8% of calcite (CaCO_3_), 24.6% of quartz (SiO_2_), 12.7% of muscovite 2M1 (KAl_2.9_Si_3.1_O_10_(OH)_2_), 11.0% of sylvite, 10.4% of barite (BaSO_4_), 2.8% of dolomite (CaMg(CO_3_)_2_), and 2.7% of orthoclase (KAlSi_3_O_8_). XRD analysis of mineral compositions of drilling fluid dried powder samples collected from a petroleum drill hole in northern Thailand showed that they contained 42.83% of quartz, 32.82% of kaolinite, 14.21 of calcite, 7.74% of albite, and 1.39% of barite (Sawaengpol and Wannakomol 2017).

Analysis of the mineralogical character of drill cuttings (C5) revealed that they contained 43.5% of calcite (CaCO_3_), 29.2% of quartz (SiO_2_), 10.1% of muscovite 2M1 (KAl_2.9_Si_3.1_O_10_(OH)_2_), 13.3% of barite (BaSO_4_), 3.4% of dolomite (CaMg(CO_3_)_2_), and 0.5% of barium chloride (BaCl_2_). XRD analysis of drill cuttings from other shales in Poland (Baltic Basin) showed that the major components of these materials are quartz, sodium aluminum dioxide (NaAlO_2_), aluminum silicate hydrate (Al_2_O_3_·2SiO_2_·2H_2_O, mineral kaolinite), and wustite (FeO) (Mykowska et al. 2015). Wilke et al. (2015) determined the mineral content in black shales in Germany and Dernmark. XRD analysis revealed that shales from the Upper Cumbrian Age contained muscovite/illite, pyrite, sanidine, orthoclase, and jarosite whereas shales from Lower Jurassic Age contained carbonate, muscovite/illite, quartz, kaolinite, albite, barite, and pyrite (Table 6).

## Conclusions

Chemical characterization of the studied drilling waste showed that they are relatively rich in Ca, Mg, and K as well as trace elements (Cu, Fe, Zn, Mn) that are essential for plant growth and thus can be used as components of soil amendment mixtures. One of the five samples of spent drilling fluids contained 270 mg kg^−1^ of Ni and exceeded the maximum permissible limit (75 mg kg^−1^) recommended by the EC regulation for safety of soil and one drilling fluid sample contained a high amount of Hg (8.77 mg kg^−1^) and exceeded the maximum permissible limit (1.5 mg kg^−1^) by almost sixfold. One of the five of drill cuttings sample (C2) contained 250 mg kg^−1^ of Pb and exceeded the maximum permissible limit (200 mg kg^−1^) recommended by the PL regulation for safety of the I class of soil. Those samples should undergo purification treatment before their use because of high levels of these elements that are toxic for humans, animals, and the environment. The heavy metal contents in the drill cuttings samples did not exceed the maximum permissible limits recommended by the EC regulation for safety of soils. The results showed the trend of higher metal contents (Mg, Cu, Fe, Mn, Zn, Al, Cr, Pb, Ni) in the drill cuttings compared to drilling fluid samples. Analysis of the mineralogical character of a solidified drilling fluid revealed that it contained calcite, quartz muscovite, sylvite, barite, dolomite, and orthoclase and of drill cuttings revealed that they contained calcite quartz, muscovite, barite, dolomite, and barium chloride. Taking the above results into account, the proper waste management, disposal, and reuse of drilling waste are vital to environment protection. The solid wastes (drilling fluids and cuttings) if properly treated can serve as raw materials for a soil amendment production. Such a soil amendment can be used for land reclamation of well sites, hard rock mining sites, abandoned coal mines, refining and smelting sites, construction sites, and other contaminated sites. Revitalization of these sites can be improved when soil amendments are used.
